# Pitaya as a New Alternative Crop for Iberian Peninsula: Cultural Practices

**DOI:** 10.3390/plants15050807

**Published:** 2026-03-06

**Authors:** Ana Rita Trindade, Pedro Matias, Vander Lacerda, Maribela Pestana, Natália Marques, Amílcar Duarte

**Affiliations:** 1 Faculdade de Ciências e Tecnologia, Universidade do Algarve, Campus de Gambelas, 8005-139 Faro, Portugal; pmmatias@ualg.pt (P.M.); fpestana@ualg.pt (M.P.); nmarques@ualg.pt (N.M.); aduarte@ualg.pt (A.D.); 2 MED Mediterranean Institute for Agriculture, Environment and Development & CHANGE Global Change and Sustainability Institute, Universidade do Algarve, Campus de Gambelas, 8005-139 Faro, Portugal; 3 CIMA/ARNET—Centro de Investigação Marinha e Ambiental/Rede de Investigação Aquática, Universidade do Algarve, Campus de Gambelas, 8005-139 Faro, Portugal; 4 Instituto Superior de Engenharia, Universidade do Algarve, Campus da Penha, 8005-139 Faro, Portugal; 5 Tropical Research and Education Center, University of Florida, Homestead, FL 33031, USA; rochalacerdav@ufl.edu; 6 Departamento de Produção Vegetal (Horticultura), Faculdade de Ciências Agronômicas, Universidade Estadual Paulista (UNESP), Botucatu 18610-034, Brazil

**Keywords:** Cactaceae, dragon fruit, irrigation, Mediterranean agriculture, pollination, production technologies, review, *Selenicereus* spp., soil fertility

## Abstract

Pitaya (*Selenicereus* spp.) cultivation has expanded in the Iberian Peninsula in recent years, driven mainly by increasing demand from the European market and by the crop’s good adaptability to Mediterranean conditions. However, the successful consolidation of this crop requires the adoption of cultural practices adapted to regional edaphoclimatic conditions and production systems. The present review aims to synthesise and critically analyse the scientific literature on pitaya cultural practices, integrating information from major producing regions worldwide and from Mediterranean environments, where data remain limited. Key topics include propagation methods for success in early development, training systems and pruning, soil management within the framework of sustainable orchard management practices and the crop’s versatility in integrating diverse agroecosystems. In addition, bibliometric analysis identified water requirements and irrigation strategies as key aspects for which region-specific guidelines are still required. This study emphasises the utilisation of floral induction techniques and the significance of supplementary manual pollination for ensuring higher productivity and superior fruit quality. Overall, this review provides a consolidated reference to support the development of sustainable and regionally adapted pitaya production systems in the Iberian Peninsula.

## 1. Introduction

In recent years, the demand for fruits with high nutritional and functional value has increased substantially. The distinctive appearance, high nutrient content, and health-promoting properties as the high antioxidant content [1,2,3,4,5] of pitaya (*Selenicereus* spp.), also known as dragon fruit, have led to its increased popularity among consumers, driving its introduction into non-traditional growing regions such as southern Europe. Despite the marked difference in climate between this region and the humid forests of Central and South America, where pitaya originates [6], the crop has shown remarkable adaptability. The cultivation of pitaya in Europe offers notable advantages for sustainable agri-food systems. Shorter transport distances between production sites and consumers significantly reduce the carbon footprint of supply chains and enhance regional economic resilience by strengthening local agricultural economies. This also contributes to improving food security and sovereignty. As a result, pitaya has become one of the most promising emerging fruit crops contributing to the diversification of agriculture in many parts of the Mediterranean Basin [7]. In the Iberian Peninsula, pitaya cultivation is still in its early stages, but it has shown remarkable growth in recent years, particularly in southern Portugal (mainly in the Algarve region) and southern Spain (Andalusia and the Canary Islands) [7,8]. This expansion is also driven by the need to adopt fruit species that require less water in response to climate change, which is manifesting as milder winters and prolonged dry seasons. In this context, the introduction of well-adapted subtropical species is a promising strategy for capitalising on emerging climatic opportunities and enhancing agricultural sustainability.

Despite the rapid expansion of this species, technical knowledge adjusted to local edaphoclimatic conditions remains scarce, and many cultivation practices are still defined empirically by producers. Most of the available agronomic information on pitaya originates from Latin America [9] and Asia [10], where environmental conditions, production systems, and management practices are substantially different from those in the Mediterranean region. Although some practices, such as propagation techniques, are broadly comparable, many others, including pollination and fruiting dynamics, remain poorly defined and require contextualisation and adaptation to the Iberian production environment.

Considering the growing interest in this emerging crop, this review compiles and analyses the key cultural practices associated with pitaya production in the Iberian Peninsula. It draws on contributions from two Portuguese research projects that aimed to understand the crop’s agronomic requirements and environmental requirements. This review emphasises production systems and key management practices, including propagation methods, training systems and pruning, soil management, irrigation and fertilisation strategies, floral induction techniques, and supplementary manual pollination. By integrating current scientific evidence with emerging regional experience, this review aims to support the development of efficient, resilient, and environmentally sustainable pitaya plantations adjusted to Mediterranean agroecosystems.

## 2. Propagation

Pitaya can be propagated in two different ways: (i) sexually, through seeds, and (ii) asexually, via cuttings. Seed propagation is primarily reserved for breeding programmes due to the high likelihood of producing genetically distinct hybrid plants [11]. In the context of commercial production, seed propagation is not recommended for pitaya plants, which are known to be heterozygous. Consequently, even crossbreeding within the same cultivar results in plants with genetic characteristics different from those of the mother plants [12].

Vegetative propagation by cuttings is the most widely adopted method for establishing pitaya plantations. This asexual propagation technique ensures the production of plants genetically identical to the mother plant, thereby preserving the cultivar’s genetic lineage. This technique involves removing cladodes from a mother plant, which, under appropriate conditions develop roots, generating new plants. The success of the technique is contingent upon the rooting capacity of the cuttings and the subsequent vigour of the propagated plants. From a commercial perspective, this method offers several advantages. It enables fruit production in less than a year [13] and promotes uniformity in plant characteristics, thereby facilitating the scheduling and execution of cultural practices, resulting in rapid economic returns.

Both intrinsic and extrinsic factors affect the rooting ability of cuttings. The intrinsic factors include the genetic characteristics of the mother plant, including its genotype/cultivar, age, nutritional and phytosanitary status. Cladode age and maturity have a significant impact on hormonal balance and the concentration of endogenous auxins, which are key hormones regulating rhizogenesis. Hight auxin/inhibitor ratio favours root formation [11]. The age of cladodes is related to their position on the mother plant. In mother plants maintained solely for propagation purposes, new cladodes are commonly allowed to develop at the basal portion of the plant, near the soil surface, as these are the first to emerge and therefore accelerate the propagation process. Those basal cladodes, characterised by a darker green colouration and exhibiting structurally tougher, generally root better in comparison to younger, light green cladodes originating from the upper portions of the mother plant. Cuttings retain the ontogenetic age of the donor plant [14]. Consequently, cladodes from older plants, which possess greater stored reserves, exhibit accelerated rooting. In contrast, younger cladodes (less than one year old) exhibit reduced rooting rates [15]. The length of cladodes selected for propagation strongly influence rooting ability as well as the number and vigour of shoots produced during the first year after planting. Cladodes measuring 15–25 cm are considered optimal for propagation [16]. The enhanced effectiveness of this process is linked with increased carbohydrate reserves and higher auxin production [17]. Collectively, these factors promote enhanced rooting success, rooting speed, and subsequent shoot development. In *Selenicereus undatus*, longer cladodes produced higher-quality root systems, regarding length, volume, and mass [18]. These outcomes were explained by an increased carbon-to-nitrogen ratio and a greater carbohydrate accumulation, both of which enhanced cutting survival. Moreover, abiotic stresses, such as nutritional deficiencies, water deficits, and suboptimal light regimes that lead to burned or etiolated cladodes tend to compromise plant vigour, resulting in weak and low-quality cuttings that are poor in photoassimilates and malformed.

Cladode health is of utmost importance, as vegetative propagation can facilitate the spread of diseases; therefore, propagation material should be selected that is free from pathogens, including bacteria, fungi, and viruses. Conversely, rigorous screening of mother plants and propagation material, combined with strict hygiene practices (e.g., disinfection of cutting tools), is essential to ensure the quality of vegetatively propagated plants [19].

To ensure optimal propagation, it is recommended that cuttings should be taken at the cladode insertion, i.e., the junction of the woody tissue with the mother plant. In instances where this is not possible, cuts may be made in the succulent portion, provided that the wound is protected against pathogen entry. This can be achieved through the application of Bordeaux paste or powdered cinnamon (*Cinnamomum verum*), as both substances act as fungistatic and bacteriostatic agents [20,21]. Cuttings should then root in pots, black polypropylene bags, or alveoli, with the bottom (basal) end buried up to 5 cm. Substrates that are well-drained and contain sand or perlite promote root development, while organic matter has been demonstrated to support subsequent growth [22].

Successful rooting is improved under conditions of 50% shade and with adequate irrigation to maintain moisture without causing waterlogging. The season of propagation affects the efficiency of rooting [16]. Colder conditions reduce the rooting rate, whereas the conditions prevailing during spring and summer exert a stimulatory effect [23]. This is likely to be a consequence of higher temperatures, which stimulate root meristem cell division and enhance cell expansion, leading to greater root growth. During the early stages of development, lateral shoots should be removed, leaving only the most upright apical shoot to promote vigorous and directed growth [24]. The application of growth regulators has been demonstrated to enhance root development and subsequent plant development. For example, dipping the cutting base in a 10 mM indole-3-butyric acid (IBA) solution for 10 s resulted in 100% rooting and improved root size [25]. Similarly, application of IBA at 6000 ppm enhanced both rooting and cladode development [26].

The growing demand for plant material to establish new plantations, in conjunction with the limited supply of plants from certified nurseries, has prompted many producers to propagate and exchange cuttings among themselves. This informal practice poses a significant risk, as the use of uncertified material can facilitate the dissemination of pathogens. In fact, the propagation by cuttings facilitates the transmission of several diseases [27,28,29], thus highlighting the dangers of unregulated propagation. The simplicity of multiplication using cuttings has resulted in less focus on alternative approaches in commercial production. In response to this challenge, recent research has focused on micropropagation as a means of enhancing disease control. Micropropagation offers several advantages, including the capacity to produce disease-free plants year-round, regardless of seasonal constraints. Although this technique entails high operating costs, owing to the requirement for aseptic laboratory conditions, highly skilled personnel, and specialised equipment, it is, nevertheless, widely used for cultivating several economically important fruit crops [30,31] and has also been successfully adapted to pitaya [32,33]. In the context of pitaya, the micropropagation procedure usually involves establishing aseptic cultures from shoot tips or lateral nodes. The in vitro plant material subsequently grows through shoot elongation, mass multiplication, and rooting [34]. However, there are still certain technical challenges to be addressed, particularly the exudation of polysaccharides and the succulent nature of tissues, which often lead to bacterial and fungal contamination or necrosis of explants [23,35,36]. To circumvent these limitations, innovative protocols have been developed. One example is the use of temporary bioreactor systems, which promote cladode proliferation in liquid culture media rather than in conventional gelled media [33].

In summary, vegetative propagation by stem cuttings remains the most practical and widely adopted method for pitaya cultivation, as it is simple, cost-effective, and allows for rapid plantation establishment. However, certain limitations, particularly the risk of pathogen dissemination and the restricted availability of certified material, emphasise the necessity of complementary approaches. Micropropagation is a valuable tool to produce high-quality, disease-free, and genetically uniform plants. Furthermore, it offers opportunities for the conservation of germplasm and the application of biotechnology, thereby strengthening the sustainability and long-term expansion of pitaya cultivation.

## 3. Production Systems

In the Mediterranean Basin, pitaya is cultivated under two production systems: (i) open-field and (ii) protected cultivation. The choice between these systems depends on local climatic conditions, production scale, and the producer’s economic objectives. Therefore, the selection of the most appropriate system should take into consideration technical, economic, and environmental factors, with the aim of achieving an optimal balance between productivity, sustainability, and profitability.

### 3.1. Open-Field Cultivation

Open-field cultivation is the most common method of producing pitayas, particularly in tropical and subtropical regions where the crop has strong traditional and economic importance and where the natural conditions are conducive to its growth. This system is characterised by lower installation costs, simpler infrastructure, and greater adaptability to small and medium-sized farms, when compared to protected cultivation. Furthermore, it facilitates the integration of pitaya into diversified cropping systems, such as agroforestry arrangements and intercropping with other fruit or perennial species [37]. Open-field cultivation exposes plants directly to natural environmental conditions, including climate and soil conditions. Planting rows arranged in a north–south orientation have been shown to maximise solar exposure, thereby promoting vegetative growth and enhancing fruit development and colouration [38]. However, this exposure also increases susceptibility to abiotic stresses, such as excessive solar radiation, heavy rainfall and high humidity, wind damage, and sudden temperature drops. High solar radiation and elevated temperatures, occurring outside the species’ typical shaded habitats, may contribute to the incidence of cladode sunburn [39]. Therefore, frost-prone areas should be avoided when establishing pitaya plantations in open fields. Moreover, irregular environmental conditions, particularly day–night temperature fluctuations, can result in delayed or uneven flowering and fruiting, leading to reduced productivity and inconsistent fruit appearance and quality when compared to protected systems.

In addition to climate, soil type is critical, as pitaya grows best in well-drained sandy-loam soils. Heavy soils and low-lying areas prone to waterlogging should be avoided. Plant establishment on raised beds improves infiltration, prevents waterlogging, and enhances soil drainage. The optimal soil pH ranges between acidic and neutral (5.5–7.0) [40,41]. Saline soils should be avoided, as they delay plant growth and development [42], and salinity levels above 4 dS m^−1^ can cause plant mortality [43]. Incorporating manure or compost into the soil is essential to increase organic matter and enhance overall soil quality. This is particularly important for this crop, given that soils in its centre of origin contain high levels of organic matter (7%) [44], much higher than the 1–2% typically found in Mediterranean Basin soils [45].

### 3.2. Protected Cultivation

Protected cultivation of this crop typically encompasses the utilisation of greenhouses, shade houses, or plastic tunnels. This production system enables greater control over important environmental factors, such as temperature, humidity, and light intensity. Therefore, protected cultivation is advantageous for producing this crop in regions with different climatic conditions to those in which it is native. By mitigating fluctuations in environmental factors, protected cultivation systems can extend the periods of vegetative growth, flowering, and fruiting periods, thereby promoting more uniform yields and often enhancing fruit quality. In the Mediterranean, during the winter season, temperatures within protected structures remain higher when compared to those in open fields. This has the effect of smoothing out the typical slowdown in vegetative growth. Consequently, plants continue to grow and produce cladodes throughout the colder months. This is particularly advantageous during the establishment stage, when rapid growth is desired, and after end-of-cycle pruning in mature plants, when the emission of new cladodes is essential to ensure fruiting in the following season. Reducing direct exposure to solar radiation, rainfall, and wind also decreases the occurrence of sunburn and mechanical injuries to cladodes and fruits.

Controlling peak temperatures effectively within greenhouses is critical during the summer months, as excessive heat severely impacts on plant physiology and crop performance. The plant is unable to adjust its temperature through transpiration, as it keeps its stomata closed during the day due to its CAM metabolism. At night, the stomata are opened and absorb carbon dioxide (CO_2_). Temperatures above 38 °C can cause severe damage to the plants and have a detrimental effect on floral induction. Therefore, effective ventilation, shading nets, and external reflective coatings are essential components of protected dragon fruit production systems and contribute significantly to improving fruit quality [39,46,47]. Nevertheless, this production system requires a higher initial investment and more advanced technical management. Economic viability depends on several factors, such as production scale, market access, and price premiums for out-of-season or high-quality fruit.

Pitaya can be cultivated under protected conditions using two main approaches: either by planting it directly in soil or by cultivating it in soilless systems. Soilless cultivation systems include hydroponics, where the roots grow directly in a nutrient solution, and substrate cultivation, where an inert or organic substrate provides physical support for the roots and is irrigated with a nutrient solution [48,49]. These systems allow precise control of the supply of water and nutrients, thereby improving resource efficiency and maintaining a favourable air–water balance around the roots. Substrate-based cultivation of pitaya has been associated with favourable vegetative growth and productive outcomes [50,51]. However, this approach can result in higher initial costs, as well as requiring more technical expertise and advanced detection systems to monitor temperature fluctuations. Using this system can also reduce soil-borne diseases, support uniform and productive crop development, and save resources through nutrient recirculation and the use of inert or recycled substrates [52,53,54,55]. These systems can operate in two distinct modes, as open or closed systems, depending on whether the nutrient solution is recirculated in a closed loop or discarded after each irrigation. Open systems are more commonly used in substrate-based cultivation, although they increase water use by up to 42% compared to closed systems [56,57,58,59]. In closed systems, drainage solution can be reused if the pathogen levels, salinity, nutrient balance, and oxygen content are properly controlled. Installing an effective drainage solution disinfection system is required to prevent the spread of pathogens [48,49].

In southern Portugal and Spain, greenhouse farming has become an increasingly important role in recent years, especially to produce berries and vegetables. The growing interest in pitaya has led several producers to include this crop initially on a small experimental scale before gradually expanding production. Through minor adaptations to the trellis structures in existing greenhouses, some farmers have successfully converted areas previously used for other crops into areas for growing pitaya. These producers quickly recognised the advantages of integrating this crop into their crop diversification strategies, as it enables continuous crop production and thus generates income throughout the year.

## 4. Training and Pruning

### 4.1. Morphological and Physiological Determinants of Pruning

Pitaya is a climbing cactus characterised by segmented, three-ribbed cladodes, which are modified stems that also perform leaf-like photosynthetic functions. These cladodes are responsible not only for photosynthesis but also for the storage of water and nutrients [7]. Along each edge connecting two faces, there are areoles, which are small, specialised axillary structures containing a bud with a multipotent meristem. From these areoles, spines, flowers, and vegetative shoots may develop. There is also a bud at the apex of each cladode, enabling its longitudinal extension.

**Figure 1:**
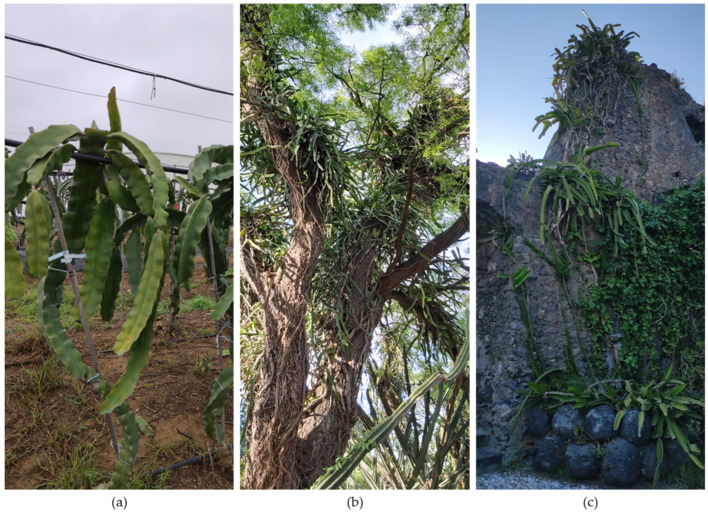
Pitaya growth and training: (**a**) commercially cultivated pitaya plants (open-field plantation in Cacela Velha, Portugal); (**b**) plants growing naturally on trees (Tropical Botanical Garden, Lisbon, Portugal) and (**c**) plants growing naturally on rocks (Norman Castle, Sicily, Italy).

From a functional perspective, pitaya cladodes can be classified into two types: (i) structural cladodes and (ii) production cladodes. Structural cladodes function primarily as axes for water and nutrient transport and provide support for the emergence of production cladodes. In a commercial plantation, they typically form a single vertical stem up to the support structure, above which the production cladodes develop (Figure 1a). Production cladodes are pendulous and constitute the structures from which fruits develop.

### 4.2. Training Systems and Support Structures

Pitaya is a perennial fruit crop with a productive lifespan of approximately 20 to 25 years [60,61]. It is characterised by a climbing growth and naturally using other vegetation, usually trees, or rocks as support (Figure 1b,c).

**Figure 2:**
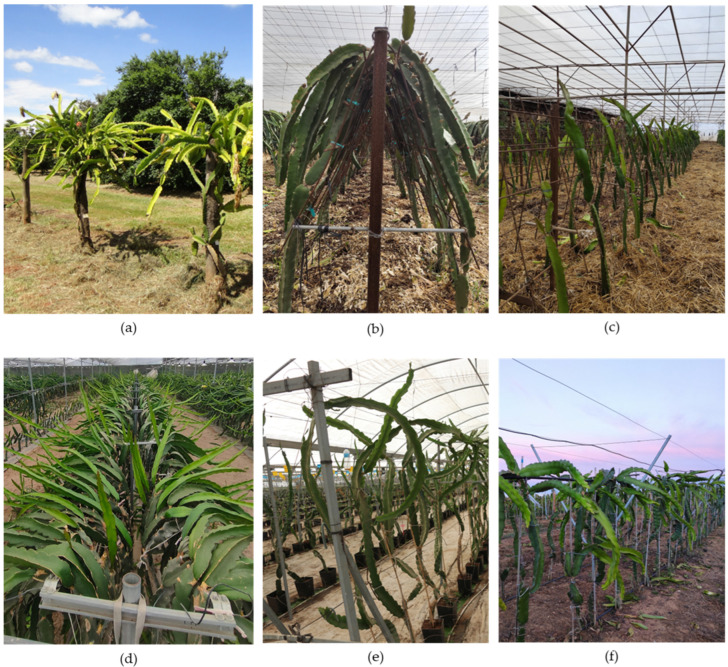
Support structures used in pitaya cultivation: (**a**) Single Post System (traditional system) (Bebedouro, Brasil); (**b**,**c**) “A-Shape Trellis” System (Tenerife, Spain); (**d**) “T Shape Trellis” System (Málaga, Spain); (**e**) vertical posts with multiple horizontal arms system, an adaptation of the raspberry training system (Cacela Velha, Portugal); and (**f**) “V Shape or Tatura System” (Cacela Velha, Portugal).

Its unique morphology and strong dependence on structural support [7] make the use of support systems necessary when cultivated (Figure 2).

The selection of support systems is primarily determined by the crop’s longevity, the cost and the maintenance requirements of the structures, and the substantial biomass load produced by pitaya.

In the countries of the Mediterranean Basin, most of these structures consist of galvanised iron posts connected by steel cables or wires, which are protected with polyethylene tubes to prevent mechanical damage to the plants.

There is an increased need to develop more innovative support structures to optimise space through higher planting densities. In addition, these novel systems aim to be more cost-effective, facilitate cultural practices such as pruning and harvesting, have a lifespan equal to or longer than that of the plants themselves, allow easy reinforcement, and support the attachment of adventitious roots. Regardless of the training system adopted, the height of the support structure (on which the productive cladodes are suspended) should not exceed 1.6 m to facilitate plantation management and harvesting operations. The training systems are classified into four types [62]: (i) Single Post System (traditional system); (ii) Trellis System, which includes the A-Shape Trellis, T-Shape Trellis, and Vertical Posts with Multiple Horizontal Arms System; (iii) V Shape or Tatura System; and (iv) Ladder System.

#### 4.2.1. Single Post System (Traditional System)

This system consists of a post, which can be made of concrete, wood, or other materials, topped with a ring or similar structure that supports the hanging productive cladodes (Figure 2a). The ring may be made of concrete, metal, a tyre, or other materials. Several authors have reported that this system achieves higher productivity and higher fruit quality, compared to trellis systems [63,64]. The difference is more pronounced in *S. costaricensis* than in *S. undatus* [63].

#### 4.2.2. Trellis System

Within trellis systems, several configurations are possible, the three most common being the following: (i) the “A-Shape Trellis” system, (ii) the “T-Shape Trellis” system, and (iii) vertical posts with multiple horizontal arms system.

In the “A-Shape Trellis” system, the main structure is made of galvanised iron and has an “A” shape, with two inclined legs (usually made of iron mesh) meeting at the top and spreading at the base (Figure 2b,c). Each A-shaped structure is installed perpendicular to the planting rows, which are double rows, i.e., consisting of two parallel lines of plants. Horizontal wires are stretched between consecutive A-shaped structures at different heights to support and train the plants. Each leg of the A-shaped structure is aligned with one of the two planting rows, allowing the plants to grow supported by both the legs and the wires.

In the T-Shape Trellis system, posts—usually made of concrete or galvanised iron—are installed, with one or two galvanised iron pipes positioned perpendicularly at the top of each pillar. Several of these pillars are placed along the planting row, and at least two wires are stretched between the pillars, passing through each end of the galvanised iron pipes (Figure 2d). Each plant is then tied to a stake until it reaches the height of the wires, providing support during growth. Above the wires, the cladodes hang down, at least one on each side, although several more may develop depending on the spacing between plants.

The vertical posts with multiple horizontal arms system are a modification of the T-shape trellis, in which horizontal arms and galvanised iron wires are installed at multiple heights, forming several layers. This system may emerge as an adaptation of the previous training system, used in raspberry cultivation (Figure 2e). For farmers seeking alternative options for small fruit crops, this adaptive trellis system is a viable solution for pitaya cultivation.

#### 4.2.3. V Shape or Tatura System

In this system, several V-shaped structures are installed in series, perpendicular to the crop row, typically made of galvanised iron, with a wire stretched between the structures (Figure 2f). In some ways like the A-Shape trellis, this system also features a double planting line, with one row of plants on each side. This configuration results in a more open plant canopy, which improves aeration and sunlight penetration.

#### 4.2.4. Ladder System

This system consists of a ladder-like structure, usually made of bamboo. Along the planting row, posts are installed, and several horizontal poles are crossed perpendicularly at different heights, creating a series of steps on both sides of each post, preferably in an alternating pattern. The plants are tied between these steps, and their cladodes become pendulous once they reach the height of the structure. This system allows for higher planting density and is both low-cost and easy to install. However, it often has a short lifespan, requires frequent replacement and is not widely used in plantations across the Iberian Peninsula.

#### 4.2.5. Live Supports

The use of living supports is particularly common in tropical and subtropical production regions. These supports generally consist of tall, slender tree species with adequate structural strength to sustain pitaya plants. Their adoption is associated with a lower environmental footprint, as no industrial resources are required for the manufacture of support materials, unlike in conventional systems. In addition, living supports enable the productive use of land through the simultaneous production of multiple outputs, aligning with the principles of agroforestry, which also aim to enhance biodiversity relative to simplified production systems [65].

Living supports can also provide habitat for beneficial insects, including pollinators of pitaya and natural enemies of key pests. In Mexico, this system is highly valued, as it makes use of existing vegetation in both smallholdings and large-scale agroforestry systems. Moreover, living supports offer natural shading, thereby eliminating the need for artificial shade nets [66]. However, adequate light penetration must be maintained to ensure optimal conditions for flowering and fruit development. Consequently, regular tree pruning is a critical management practice to control canopy architecture and promote sufficient light incidence on the pitaya plant. In the Iberian Peninsula, this system remains uncommon, although its adoption is technically feasible and may offer potential benefits under suitable management and climatic conditions.

### 4.3. Objectives of Pruning

Pruning is defined as the removal of plant parts, such as branches, roots, or buds, when such intervention influences the plant’s physiological response [67]. It is an ancient cultural practice, documented since the time of Ancient Greece [68,69], and today it remains a fundamental management operation in the maintenance of many perennial crops [70,71,72]. For this reason, pruning is an essential cultural practice in the management of many high-value crops.

The main objectives of pruning in pitaya are closely linked to its unique growth habit and physiological characteristics, aiming to optimise plant development, fruit production, and overall crop management [73]. The specific objectives can be summarised as follows: (i) plant formation; (ii) growth control and canopy maintenance; (iii) stimulation of flowering and fruiting; and (iv) facilitation of pest and disease management.

Pruning facilitates canopy management, making various cultural practices easier and reducing production costs. Properly distributed cladodes, without excessive overcrowding, enhance air circulation and light penetration, facilitate natural pollination, and improves access for harvesting. In addition, when agrochemicals are required, their application becomes more efficient as the inner parts of the canopy are more accessible.

In the absence of pruning to regulate vegetative growth, pitaya plants continue producing new cladodes throughout most of the year. Excessive vegetative development diverts metabolic resources away from reproductive processes, reducing both flowering and fruit set. Pruning helps limit the number of cladodes, promoting the formation of thicker, well-nourished ones that are physiologically more capable of supporting abundant flower and fruit production.

### 4.4. Types of Cuts

Pruning cuts can be classified into three main types: (i) heading cuts, (ii) reduction or drop-crotch cuts, and (iii) thinning cuts. Heading cuts consist of removing part of the cladode at any point along its length to stimulate the formation of new cladodes from the lateral buds below the cut. A reduction or drop-crotch cut consists of removing an older cladode just above a younger lateral branch, stimulating the growth towards the remaining cladode. A thinning cut consists of removing a cladode by cutting it at its base, allowing the complete removal of an undesired cladode. When the heading cut removes only the terminal portion (tip) of the cladode, the operation is referred to as tipping.

### 4.5. Pruning Types

#### 4.5.1. Formative Pruning

The purpose of formative pruning is to guide the plants according to the training system adopted and is performed during the first years of the plant’s life, from the nursery stage until it reaches its mature size.

The main objectives of formative pruning in pitaya are as follows: (i) to establish a strong and healthy structural framework of cladodes that can support future production cladodes and fruit loads, even under challenging environmental conditions; (ii) to prevent production cladodes from developing too close to the ground; (iii) to optimise the distribution of production cladodes, avoiding competition for space, light, and resources; and (iv) to manage the future canopy structure so as to maintain minimal structural cladodes while promoting aeration and sunlight exposure of the production cladodes.

Formative pruning aims to promote the development of cladodes that, due to their position and vigour, will constitute the plant’s structural framework.

In this crop, regardless of the training system adopted, plant formation involves maintaining a single, unbranched vertical main stem up to the height defined by the training system (usually 1.5–1.6 m). Above this height, the cladodes are guided to hang down. Although in some plantations more than one main stem may be used, this practice remains uncommon.

**Figure 3:**
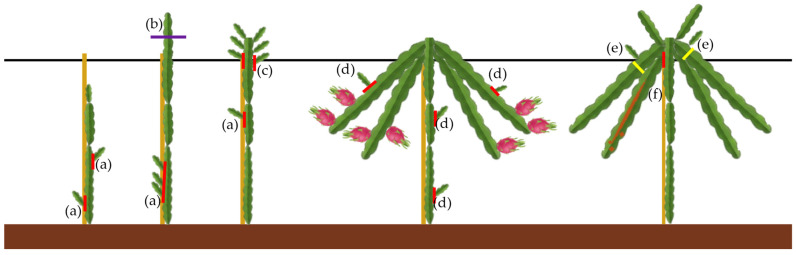
Types of cuts and pruning operations in pitaya. Red lines represent thinning cuts; purple lines represent heading cuts; and yellow lines represent reduction or drop-crotch cuts. Formative pruning: (**a**) removal, through thinning cuts, of cladodes that are not part of the desired structure; (**b**) heading cut of the main cladode slightly above the support; (**c**) removal, by thinning cuts, of shoots not selected to become productive cladodes. Maintenance pruning: (**d**) removal, by thinning cuts, of cladodes that are neither structural nor productive; (**e**) selection of shoots at the base of the productive cladodes after fruiting, to replace them in the following season, performed through reduction (drop-crotch) cuts; and (**f**) removal of diseased cladodes through thinning cuts.

Formative pruning should begin at the nursery stage, where plants should be maintained with a single main stem, and should continue after planting. Until the support structure height is reached, lateral cladodes emerging from the main stem should be removed through thinning cuts (Figure 3a). When the uppermost cladode reaches the height at which it should hang, two pruning operation can be applied: (i) a heading cut made approximately 10–15 cm above the support structure, which promotes the emergence of new cladodes below the cut (Figure 3b); or (ii) bending the portion of the cladode above the support structure downward so that new cladodes emerge at the upper side of the bend, which should likewise be guided to hang. Among the newly emerged cladodes, a selection should be made of those that are well-distributed and oriented in multiple directions. The number of cladodes chosen should be adjusted according to plant spacing and the training system employed. Cladodes that are not selected should be completely removed through thinning cuts (Figure 3c). New productive cladodes can be selected by choosing shoots from the base of old productive cladodes, and their growth should be stimulated through a reduction or drop-crotch cut (Figure 3d).

#### 4.5.2. Maintenance Pruning

The main purpose of maintenance pruning is to preserve a balanced number of productive cladodes, ensuring that the canopy remains well-ventilated, adequately illuminated, and free from excessive crowding. An excessive number of tangled cladodes negatively affects fruit size, resulting in smaller fruits compared to those produced under more open canopies, without providing any increase in overall yield [74].

The main objectives of maintenance pruning in pitaya are as follows: (i) to control excessive vegetative growth and prevent canopy overcrowding; (ii) to maintain a balanced relationship between vegetative development and fruit production; (iii) to renew productive cladodes by removing aged, unproductive, or poorly positioned ones (Figure 3f); (iv) to improve light penetration and air circulation within the canopy, thereby reducing the risk of pests and diseases; (v) to preserve the structural framework of the plant and maintain the training system; (vi) to enhance flower induction and fruit set; and (vii) to improve fruit quality and size by directing resources to well-positioned, well-sized cladodes.

Maintenance pruning should be carried out throughout the year with at least two specific stages occurring during the production cycle. The first stage should take place approximately two months before the first flowering flush, and the second stage should begin about two weeks after harvest (Table 1).

Shoots emerging from productive cladodes should be removed to prevent unnecessary sink organs from consuming photoassimilates. This type of pruning requires special attention in the period approximately two months before the first flowering flush. However, if these new shoots have begun to grow during the current vegetative cycle and are well positioned, they may serve as potential productive cladodes in the next cycle (Figure 3e). Therefore, they should be retained, and the productive cladode should be removed later, after fruiting (Figure 3d).

After harvest, the focus of pruning should be on the removing the oldest cladodes, those with limited light exposure, and those that were highly productive during the previous season.

Throughout the year, any unproductive, poorly positioned, or weak cladodes should be removed, as should any developing shoots that emerge on structural or productive cladodes and will not be used in the future, for either structural or productive purposes (Figure 3d). This includes those emerging on the main stem (Figure 3a). This operation should be carried out as soon as possible after the shoots emerge.

#### 4.5.3. Floral Induction Pruning

Floral induction pruning is typically carried out before the floral induction period (March–April) and involves the removal of the apical portion of productive cladodes that are still developing, through a heading cut (often referred to as tipping). This specific cut disrupts apical dominance, thereby stopping the apical growth of the cladode [75,76]. The cut reduces the rate of sap circulation, causing its retention. Without this intervention, the sap would otherwise remain in rapid circulation and be used for cladode growth [77]. The retention of sap within the cladode promote flowering by stimulating reserve accumulation in these cladodes, thereby promoting flowering, fruit set, and fruit quality [67].

### 4.6. Pruning Tools and Disinfection

The process of pruning entails the use of pruning shears, which can be manual or electric. These tools are relatively inexpensive (with electric models being more costly) and easy to maintain. However, they do require regular maintenance to ensure that the cutting blade is sharp, the joints are adequately lubricated, and, for electric shears, the batteries are properly charged. In thinning operations, when the cladodes to be removed are very young (up to 5 cm long), they can simply be removed by pulling them out manually, with the hand protected by a glove.

To prevent pathogen transmission between plants, pruning tools must be routinely disinfected [78]. In nurseries, tools should be sanitised, ideally between each plant. In field conditions, tools should be sanitised at least when moving between planting lines, plots, or farms. Whenever healthy and diseased plants are present in the same plot or farm, the healthy ones should be pruned first, with the diseased plants being pruned subsequently and ensuring thorough disinfection afterward.

The employment of chemical products for the purpose of tool disinfection demands specific precautions, making it essential to wear gloves, goggles, and, when indicated, other personal protective equipment. Prior to the disinfection procedure, it is important to remove debris, such as plant and soil residues from pruning tools, to ensure the disinfectant works effectively. Disinfectants can act either bacteriostatically or fungistatically by inhibiting pathogen proliferation, or have a direct lethal action against fungi, bacteria, and viruses [79]. Commonly used products include bleach, alcohols, chlorine dioxide, and hypochlorites. Each of these products have a distinct set of advantages and limitations. Bleach is inexpensive, widely available, and has a broad spectrum of action, including viruses. However, it is corrosive to metal, requiring rinsing afterward and the use of lubricant to protect tools. Alcohol is less corrosive and acts rapidly against bacteria and fungi, although its effectiveness against viruses is limited [80].

### 4.7. Pruning Waste Management

**Figure 4:**
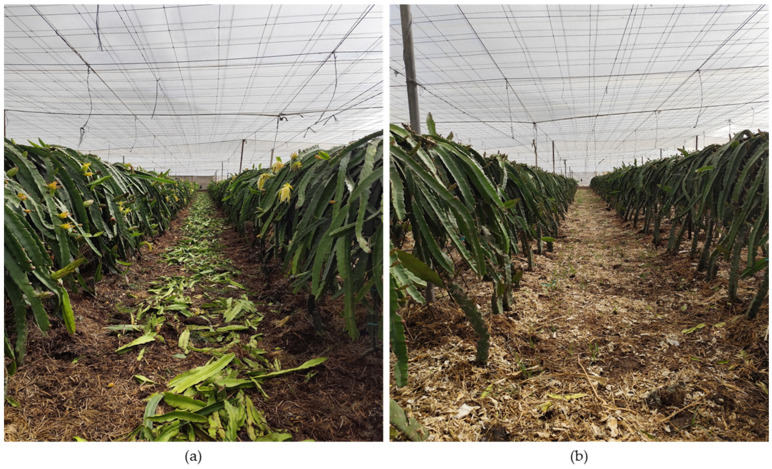
Pruning waste management in a protected pitaya production system using the shredding of pruning residues: (**a**) pruned cladodes placed in the inter-rows for drying and (**b**) cladodes after drying and shredding (Tenerife, Spain).

Compared with other crops, the management of pitaya pruning residues requires special care, as cladodes can easily take root when in contact with soil. In soil-based plantations, cladodes can be left to dry before being shredded and incorporated into the inter-row area (Figure 4a,b). In soilless systems, the disposal of cladodes must be carried out responsibly, as improper discard in the environment can lead to unwanted rooting.

Diseased cladodes also require careful disposal; they should not be incorporated into the soil without prior treatment. One possible solution is to compost the cladodes together with other agricultural residues [81], which are often available on the farm or from neighbouring farms [82]. This approach can be applied both in soil-based plantations and in soilless production systems, allowing the resulting compost to be reused as mulch within the rows or as an organic amendment in the inter-rows of pitaya plantations, as well as in other plantations, or even vegetable gardens. When applied to the soil, compost increases soil organic matter content and contributes to long-term soil fertility [83]. Composting offers yet another important advantage for the sustainable management of pitaya pruning waste, as it can eliminate pathogens through the heat generated during the process or via biological control by antagonistic microorganisms that develop in the compost [84,85].

## 5. Soil Management

Weed control remains one of the main challenges in irrigated plantations. This is particularly true for open-field pitaya cultivation, where effective soil management is essential for maintaining productivity [86]. Pitaya provides limited shade of the soil surface, which in turn enhances soil warming and promotes weed seed germination and growth. Furthermore, as both pitaya and most weed species have relatively shallow root systems [87,88], there may be intense competition for water and nutrients in the upper soil layers.

Besides chemical control and manual control, which, although effective, is highly time-consuming and labour-intensive [89], several other strategies can be used for weed control: (i) cover crops; (ii) mulching; and (iii) livestock integration. These approaches provide multiple agroecosystem benefits, such as improving soil quality and fertility, enhancing nutrient cycling, increasing soil water retention, and offering protection against soil erosion, among others.

### 5.1. Cover Crops

**Figure 5:**
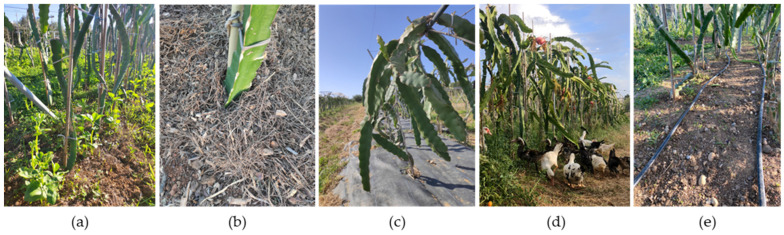
Soil management strategies: (**a**) early development of faba bean (*Vicia faba*) used as a cover crop in the planting row; (**b**) mulching with grass clippings and garden pruning residues; (**c**) black polyethylene screen used as mulching; (**d**) livestock (ducks) integration; and (**e**) soil six months after livestock integration.

In Brazil, it is common practice in open-field pitaya plantations to sow forage peanuts (*Arachis pintoi* Krapov. & W.C.Greg) alongside grass species such as *Brachiaria* spp. and *Avena strigosa* L. (black oat) [90]. This cover crops effectively suppresses weeds while simultaneously enhancing nitrogen fixation and biomass accumulation. After the living cover is mechanically mowed, the resulting biomass is typically left on the soil surface as mulch or incorporated into the soil, further contributing to organic matter enrichment and improved soil fertility. However, this approach has not yet been so widely implemented on large-scale commercial pitaya production systems in Mediterranean countries, being predominantly practiced within smallholder contexts (Figure 5a). The introduction of other legume species with either similar or different growth habits and greater adaptation to local soils and climate, such as faba bean (*Vicia faba*) or hyacinth bean (*Lablab purpureus*), could represent a viable alternative, especially in open-field growing situations. Notably, some of these species are already used as cover crops specially in organically managed citrus orchards, suggesting their potential success in pitaya cultivation. However, while living covers actively contribute to soil fertility and ecosystem balance [91], their success requires careful management, particularly under water-limited conditions [92].

### 5.2. Mulching

Mulching can be implemented using two main categories of materials: (i) organic materials, such as plant residues and other organic matter (Figure 5b), and (ii) synthetic material, such as weed-control fabrics (Figure 5c). Compared to cover crops, mulching provides a more passive yet highly efficient strategy for achieving similar agronomic objectives and may be preferred in situations where a simpler, low-maintenance management option is desired. It is widely recognised as an effective and practical strategy to control weeds, while also retaining soil moisture, reducing surface evaporation, and moderating soil temperature [93]. Organic mulching is becoming increasingly relevant in the context of global efforts to enhance water-use efficiency in agriculture, particularly as it can be implemented using a wide range of organic by-products, thereby promoting the circular utilisation of agricultural residues [34,67]. Organic mulch also enhances soil fertility and biological activity by improving nutrient cycling, stimulating soil enzymatic processes, and fostering diverse and active microbial communities [94], in addition to contributing to soil carbon sequestration [45]. These positive outcomes are closely linked to the accumulation of organic matter in the soil [95], a factor of particular significance in Mediterranean regions, where low levels of soil organic carbon are a common limitation to fertility and long-term productivity [96,97].

Besides organic mulching, synthetic materials, such as black polyethylene screens, are widely used, particularly in intensive or protected cultivation systems. This plastic film is a more durable, low-maintenance alternative to mulch and is one of the most effective methods of weed suppression in fruit crops, as it virtually eliminates weed growth [98]. Consequently, the need for chemical or mechanical weed control methods is greatly reduced. This practice can also improve plant health, enhance nutrient uptake efficiency, decrease water demand [99] and promote vigorous vegetative growth [100]. However, continuous long-term use of plastic screens in the long term may limit soil aeration and microbial activity, reducing soil fertility and biological health. This is due to the physical barrier preventing the incorporation of organic residues that would otherwise contribute to soil renewal. In hot climates, excessive soil heating beneath the screen can also cause root stress, particularly in crops with shallow roots, such as pitaya. Polyethylene is non-biodegradable, and its incorrect disposal or recycling can contribute to plastic and microplastic pollution [101,102], negatively impacting the environment and human health [103,104] as well as increasing labour and material costs.

### 5.3. Integration of Livestock

Although not as common as other practices, the integration of livestock into soil management represents a sustainable way to control weeds and cycle nutrients. This approach reduces the need for external inputs, including chemical, organic and biological fertilisers. In fact, many organic and biological-based fertilisers are themselves derived from recycled plant and animal by-products [105], aligning with the concept of a “circular nutrient economy”. This concept involves recycling and reintegrating agricultural residues back into production systems [106], thereby reducing dependence on finite mineral resources and promoting more sustainable nutrient management. In pitaya plantations, integrating poultry species, such as chickens and ducks (Figure 5d), offers a promising agroecological strategy that combines weed control and soil improvement [107]. Allowing these animals to forage on spontaneous vegetation can substantially reduce weed pressure, either by consuming herbaceous species or by trampling them (Figure 5e). This practice reduces labour requirements and minimises or even eliminates the need for mechanical or chemical weed control. Their natural behaviour of scratching and grazing disturbs weed seedlings, while the manure they deposit enriches the soil with nitrogen, phosphorus, and micronutrients [108], increasing nutrient cycling.

To ensure this practice is effective in the long term, careful management of stocking density, grazing duration and seasonal timing is required. High stocking densities of poultry can lead to soil compaction and damage to the shallow roots of pitayas, whereas rotational or low-density grazing maximises agronomic benefits without compromising the structure of plantation. Stocking densities should be adjusted according to both management objectives and the seasonal growth cycles of weeds and the main crop. Poultry-based weed control tends to be most effective at certain times of the year, particularly after rainfall, when young weeds are more palatable. In newly established plantations, poultry may break or peck at tender cladodes. In such cases, physical protection or temporary exclusion is recommended. During the fruiting period, overgrazing should be avoided as, once weeds have been suppressed, the animals may turn their attention to the ripe fruits. Furthermore, the direct contact with the soil can increases the risk of zoonotic pathogens or parasites, making animal health and hygiene essential prerequisites for using poultry.

Poultry can also help to control small invertebrate pests, such as snails, which can damage young cladodes. Moreover, they can enhance functional biodiversity by attracting beneficial insects and supporting biogeochemical cycling. In the long term, continuous grazing by poultry helps to reduce populations of herbicide-resistant weeds by limiting seed production and reducing the selective pressure exerted by persistent species. In addition, poultry can complement living cover systems by controlling excess biomass and stimulating nutrient cycling [92] without fully removing soil vegetation. As well as providing these ecosystem and agronomic services, poultry can also generate valuable products, such as eggs and meat. This can diversify farm income, particularly for smallholders, and on the other hand can be more eco-efficient, particularly if efficient farming practices, local feed sourcing, and effective nutrient recycling are ensured [109].

This alternative form of soil management aligns with the principles of integrated plantation management by harnessing natural ecological synergies. It is also compatible with integrated crop–livestock systems, which combine cultivation and livestock production to optimise the use of space and time [110,111]. In Mediterranean and semi-arid regions, such integration offers an effective means of optimising the use of key local resources, such as water, thereby enhancing land use efficiency and productivity and contributing to the long-term conservation of soils and agroecosystems [112].

## 6. Irrigation

The CAM metabolism of this crop results in higher water-use efficiency compared to C_3_ plants [88,113,114]. Whereas C_3_ plants typically lose approximately 600 H_2_O molecules per fixed CO_2_ molecule, CAM plants lose only about 10 H_2_O molecules per molecule of CO_2_ assimilated [94,95]. An additional advantage is the lower stomatal frequency in CAM plants, approximately 2500 stomata per cm^2^ compared to 20,000 stomata per cm^2^ in C_3_ plants [115]. In addition to CAM metabolism, this crop stores water in its cladodes, maintaining physiological activity during extended droughts [116,117]. The absence of true leaves and the presence of a waxy surface (in some cultivars) reduce transpiration and reflect solar radiation, thereby enabling survival during prolonged periods of high temperature and limited water availability [118].

Despite all these water-saving mechanisms, pitaya typically grows in semi-humid tropical forests, where the annual rainfall varies between 1700 and 2540 mm [60]. In the Iberian Peninsula the precipitation regime differs, with rainfall (500–700 mm) concentrated in the cold seasons (autumn and winter) and dry conditions during the warm seasons (spring and summer) leading to a high-water vapour pressure deficit in the atmosphere [119,120]. Therefore, cultivating this crop in the Mediterranean requires irrigation to offset crop evapotranspiration, which cannot be sustained by the prevailing environmental conditions [121]. This makes irrigation indispensable for sustaining growth, flowering, fruit quality and achieving commercially viable yields [122]. Even so, much less water is used to irrigate pitaya plants than most other fruit crops [123]. In a scenario of water scarcity where it may be necessary to suspend irrigation for extended periods, pitaya is able to survive, although its production is reduced. Therefore, implementing this crop on a farm alongside other crops can allow the grower to better adapt their water management strategy in case water supply is limited.

As pitaya cultivation does not generally require irrigation in its native environment and is a relatively recent crop in Mediterranean agriculture, information regarding its water requirements remains limited. One of the few quantitative studies on pitaya irrigation reported that, under the arid conditions of Israel, pitaya requires approximately 1200 to 1600 m^3^ ha^−1^ yr^−1^ of water to sustain yields ranging from 30 to 40 t ha^−1^ [88]. Based on these values, the crop’s water productivity (WP) can be estimated at about 25 kg m^−3^. In contrast, C_3_ fruit crops such as avocado and citrus, cultivated under the same arid conditions of the Negev Desert, exhibit considerably lower WP values, averaging approximately 1.6 kg m^−3^ for avocado and 5.2 kg m^−3^ for citrus. These differences highlight the remarkably higher water-use efficiency (WUE) of pitaya and suggest that similar patterns are likely to occur in other Mediterranean regions. Nevertheless, due to the current lack of standardised irrigation guidelines tailored to Mediterranean conditions, site-specific water requirements should be estimated following the FAO56 methodology. This approach integrates local reference evapotranspiration (ET_0_) with the appropriate crop coefficient (Kc) [124] to calculate crop evapotranspiration (ETc), thereby providing an accurate estimate of the crop’s actual water demand. ETc-based irrigation scheduling can then be adjusted to local agronomic conditions, including plant height, canopy density, training system, and soil cover. Despite this, Kc values for pitaya have not yet been standardised for different environmental and management contexts. Existing references remain limited to a few recommendations, suggesting that Kc values may range from 0.6 to 0.9 for planting densities above 2000 plants per hectare [125]. Field-based determinations further indicate that, in young pitaya plantations (<3 years old), Kc may vary between 0.45 and 0.71, depending on growth stages [126,127]. However, these values were obtained under highly specific experimental conditions and therefore do not accurately reflect the diversity of management practices currently adopted in commercial production systems.

Regarding water quality, several studies evaluating the response of pitaya to saline conditions suggest that it exhibits low to moderate salinity tolerance, particularly during the early stages of development. Some authors found that irrigating young *S. undatus* plants with water ranging from 0 to 4 dS m^−1^ resulted in substantial reductions in shoot and root growth above 2 dS m^−1^, as well as approximately 50% plant mortality at 4 dS m^−1^ [128]. Similar results were observed when irrigation water at 5 dS m^−1^ caused pronounced decreases in root length and secondary cladode formation [129]. Germination and seedling development studies [105] have shown that salinity levels above 2.0–2.5 dS m^−1^ markedly reduce germination rates and early biomass accumulation, further corroborating the species’ pronounced sensitivity to salt stress [130]. Under salt stress conditions, red pitaya plants increase their levels of proline, amino acids and total sugars [131], reflecting typical osmotic adjustment responses that help mitigate cellular damage, as reported in other species [132]. Taken together, these findings suggest that, although mature, field-grown plants may tolerate slightly higher salinity levels under certain environmental and management conditions, a conservative irrigation water threshold of 1.5–2.0 dS m^−1^ should be considered until region-specific studies establish localised guidelines for Mediterranean plantations.

In Mediterranean conditions, localised irrigation systems, such as drip irrigation, are the most suitable, ensuring precise water application and allowing fertigation. Combining drip irrigation with mulching techniques can increase water-use efficiency further, as mulching material can minimise water loss through evaporation. It is recommended to maintain moderate and consistent soil moisture, avoiding both extended drought and waterlogging. In fact, as with other subtropical fruit species, such as avocado, pitaya is extremely sensitive to waterlogging. Excessive irrigation or inadequate soil drainage greatly increases the risk of fungal diseases, particularly *Phytophthora* and *Fusarium* [73], and raises the likelihood of flower bud and young fruit abscission. Based on the current evidence, pitaya is one of the most water-efficient subtropical fruit crops suitable for Mediterranean agriculture. However, establishing region-specific irrigation requirements and salinity tolerance limits remains a priority for future research.

## 7. Fertilisation and Plant Nutrition

The high adaptability of pitaya to various growing conditions allows the use of soils less suitable for more demanding crops such as other traditional fruit plants. In its natural environment, pitaya is considered undemanding in terms of soil fertility and has low macronutrient requirements. In this situation, these plants, which have an accentuated vegetative vigour and are not pruned, survive and grow quite well, but fruiting is not very common. These are plants that respond very positively to fertilisation and water availability when cultivated commercially. They benefit significantly in terms of productivity from the incorporation of organic matter into the soil and from a balanced fertilisation plan adapted to each phase of the cycle [133].

Sustainable nutrient management in pitaya requires an integrated approach that balances mineral fertilisation with organic amendments to maintain soil health, and long-term productivity [134] integration of soil chemical analysis with the nutritional monitoring of shoots (cladodes) enables a transition from generic fertilisation to precision management, which is essential for the economic viability of pitaya cultivation.

The pitaya nutrient requirements are highly age-dependent and vary by species, with different requirements at different life stages. Nitrogen (N), phosphorus (P), and potassium (K) are the primary drivers of growth and yield, with K being particularly critical for enhancing fruit quality, sugar content, and post-harvest shelf life [135,136]. The nutrient absorption rate of the *Selenicereus undatus* (Haw.) species was studied under greenhouse conditions. After one year of cultivation, nutrient accumulation in the aerial part (cladodes) followed a descending order for macronutrients: K > calcium (Ca) > N > magnesium (Mg) = P > sulphur (S). For micronutrients, the order was as follows: zinc (Zn) > manganese (Mn) > iron (Fe) > boron (B) > copper (Cu). Regarding nutrient export by the fruits, the order was K > N > P = Mg > Ca > S > Zn > B > Fe > Mn > Cu or K > N > P > Ca > Mg > and Mn > Fe > Cu > Zn > B [137]. Furthermore, the timing of application is critical for optimising fruit quality and export. The highest requirements for P, K, Ca, Mn and B occurred between 180 and 240 days after planting, while N, Mg and S were higher between 120 and 180 days. Furthermore, Zn demand was highest between 300 and 360 days, whereas Cu and Fe requirements remained constant throughout the experiment [138,139].

Fertilisation strategies must consider the plant’s unique CAM physiology, which often results in a slower response to N application than is seen with other cacti. The latter require approximately 12–13 weeks to increase net CO_2_ uptake [117]. Stem N levels increased to 0.9% when 0.16 mM N was applied, rising to 2.5% as the concentration increased to 16 mM N [117]. The chlorophyll content per unit area also increased substantially from 0.30 to 0.63 g m^−2^, in the presence of 16 mM N [117]. Based on these results, it was suggested that using an optimal ratio of N-P-K-Mg, such as 9.6: 4.8: 17.6: 2.4, in combination with organic compost could promote healthy plant growth and prevent physiological disorders like fruit splitting.

In *S. undatus* and *S. monacanthus*, K application resulted in higher crop yields and improved fruit quality. The estimated K dose required to maximise fruit production in these two species was 120 g K_2_O per plant in the first year, increasing to 200 g K_2_O in the second and third years, alongside a fixed nitrogen dose of 100 g N per plant [140].

A strategic approach to fertilising with N and K improves the nutritional and sensory profile of the fruit and mitigates the fertility constraints of limestone-rich agricultural areas. Research by Chen et al. [141] shows that NK synergy can significantly boost fruit nutrient concentrations, raising N, P and K levels by 33.7%, 31.3% and 35.5%, respectively, while optimising the absorption of Mg, Zn and Se in *S. undatus* cultivation. K plays a pivotal role in determining fruit quality by increasing soluble sugar levels and the sugar-to-acid ratio, thereby reducing titratable acidity. However, balanced management is essential, as excessive K can hinder the absorption of Ca and Mg.

Soil liming, fertigation, or foliar application with silicon (Si) can help to control fungal diseases in pitaya plants. A study in Malaysia [142] found that plants that received 5.0 mL of Si per litre applied to the soil showed lower disease incidence and severity, as well as greater Si accumulation in cladodes and fruits. Therefore, Si application could be an effective alternative in the management of pitaya diseases, while improving fruit production and mitigating the effects of abiotic stress. This practice produces low levels of chemical residue in the fruit and reduces environmental pollution caused by intensive application of fungicides.

Furthermore, precise micronutrient supplementation, especially boron (B) and calcium (Ca), is fundamental for reproductive success and fruit structural integrity. B is essential for pollen tube germination and fertilisation, directly impacting fruit set and size, while Ca plays a vital role in cell wall stability and reducing physiological disorders. According to Sahu et al. [143], foliar application of 300 mg L^−1^ of B to 7-day-old flower buds maximises pollen germinability, fruit weight, and pulp firmness in *S. monacanthus*. These improvements are driven by the upregulation of carbohydrate-metabolising enzymes (invertase and sucrose synthase) and the simultaneous inhibition of cell wall-degrading enzymes (cellulase and polygalacturonase), which preserves the fruit’s structural integrity. Furthermore, this treatment significantly enhances the nutritional and bioactive profile of the fruit, increasing the levels of total soluble solids, protein, and ascorbic acid, betacyanin, and flavonoids. Such findings highlight the importance of B supplementation in optimising dragon fruit quality, particularly in nutrient-deficient acidic soils.

While N promotes healthy canopy growth, P and K are essential for floral induction and bud development. They also enhance nectar quality, attracting nocturnal pollinators of *Selenicereus* spp. [144]. Furthermore, mineral nutrition interacts with photoperiodic and temperature regulations to determine the flowering season’s duration and the intensity of multiple flowering flushes (4–7 per year). Therefore, implementing integrated agronomic techniques, including optimised fertilisation schedules, is vital to ensure high floral anthesis rates and minimise flower abscission in commercial plantations [144].

Additionally, the incorporation of organic fertilisers, such as well-decomposed manure or compost, improves soil structure and water-holding capacity and enhances the bioavailability of essential micronutrients [134]. Recent findings also indicate that organic compost and sheep manure attenuate the harmful effects of salinity on red pitaya seedlings [131].

As expected, a moderate to strong positive correlation was observed between soil and shoot nutrient levels and the final concentrations of nitrogen (N), phosphorus (P), and potassium (K) in the fruit pulp, based on experiments conducted in twenty *S. costaricensis* plantations [145].

Recent research [134] highlights the effectiveness of high tunnel systems and organic amendments in optimising the growth and nutritional profile of various pitaya species. Under high tunnel conditions, *Selenicereus* species (particularly the yellow pitaya) demonstrated higher survival rates and an absence of disease compared to open-field cultivation, which suffered from fungal and bacterial infections. By 365 days after plantation, the order of nutrient accumulation in the shoots was as follows: K > Ca > N > Mg > P > S > Fe > Zn > B > Mn, with plants grown in high tunnels showing significantly higher concentrations of N, K and Fe. Furthermore, this study suggests that an application rate of 25 t ha^−1^ of vermicompost is optimal for enhancing soil fertility and nutrient solubility, while avoiding the salinity stress and nutrient antagonism observed at higher doses. Spectral reflectance analysis confirmed these findings: plants treated with the correct organic rates exhibited increased near-infrared (NIR) reflectance, which is a key indicator of improved leaf structure and higher chlorophyll content. These results highlight the importance of species-specific fertilisation strategies combined with protected cultivation environments for maximising the sustainable productivity and nutrient density of the fruit.

According to Mizrahi [51], the stomata on the surface of the fruit also function via the CAM pathway, influencing transpiration and post-harvest shelf life. Based on this, plant growth regulators can complement mineral nutrition: for example, while gibberellic acid (GA_3_) delays flowering, cytokinins can induce it. These findings emphasise that successful fertilisation depends not only on nutrient supply but also on an understanding of shallow root physiology and how plants respond to environmental conditions through their hormones.

## 8. Floral Induction Techniques

### 8.1. Fundamental Factors Affecting Floral Induction

Flower induction in pitaya is strongly influenced by environmental conditions, the physiological status of the cladodes, and the cultural practices applied throughout the production cycle. Although this topic was recently reviewed by Dhiman et al. [144], most of the available knowledge derives from studies conducted in tropical and subtropical regions. These conditions differ markedly from those found in the Mediterranean, particularly with respect to temperature, solar radiation, and photoperiod. As pitaya cultivation expands across the Iberian Peninsula, a comprehensive understanding of the mechanisms underlying floral induction, as well as the identification of effective strategies to optimise flowering, becomes essential to ensure consistent yields and economically viable production.

Together, photoperiod, temperature, and light availability determine the environmental window in which pitaya can flower. However, the transition of a cladode to reproductive growth also depends on its physiological status, creating an important interaction between external cues and internal capacity.

Pitaya produces flowers only on cladodes that have reached adequate developmental maturity, which typically occurs when they are 8–12 months old. However, this timeframe is strongly affected by management practices and growing conditions. In protected cultivation, where continuous vegetative growth is sustained throughout the year, young cladodes can initiate their first flowering, albeit with lower floral potential, if environmental conditions are suitable and the cladodes are sufficiently mature [146].

For successful induction, cladodes must also display sufficient physiological competency. This means they must be structurally robust and free from biotic (pests, diseases) and abiotic (sunburn, dehydration) stresses and injuries. They must also possess a functional vascular connection to the main stem. A key factor in this physiological competence is the accumulation of carbohydrate reserves, since mature cladodes function simultaneously as both photosynthetic organs and as storage tissues. Adequate reserves, particularly starch, which represents the primary carbon storage form in pitaya [147], are essential for meeting the energetic demands of floral initiation, bud development, and subsequent fruit growth. The transition from vegetative to reproductive development is therefore closely linked to the carbon–nitrogen (C/N) balance within the cladode [148]. High nitrogen availability favours vegetative expansion and delays flowering [149], whereas greater carbon accumulation, along with associated changes in sugars and hormone distribution, promotes reproductive differentiation [150]. Therefore, even when external environmental conditions are favourable, flowering will only occur when cladodes have accumulated sufficient carbon reserves and have reached a metabolic state conducive to reproductive development.

Finally, position and cladode size are important factors: cladodes located in well-illuminated sections of the plant show greater floral potential, reflecting higher rates of carbon assimilation compared to shaded or internal cladodes. Cladode size is also a relevant predictor of reproductive capacity: larger, thicker cladodes (typically identifiable by their more intense green colouring) have greater storage of carbohydrates and water, supporting the energetic demands of flower and fruit development. Very thin, small, or newly formed cladodes usually remain strictly vegetative. Growers should prioritise maintaining and developing well-formed cladodes measuring 40–60 cm, since cladodes within this size range show the highest probability of flowering and produce a greater number of fruits per cladode [151]. In contrast, the cladodes measuring 20 cm or less should be removed, as they have a markedly reduced likelihood of flowering and produce inferior quality fruit when they do flower [152].

### 8.2. Photoperiod Manipulation

Pitaya flower induction is influenced by daylight length [153] and occurs under long-day conditions, when the photoperiod approaches or exceeds a critical threshold of around 12 h [154,155]. The progressive increase in daylight hours during late spring in the Northern Hemisphere contributes to floral induction, but this is rarely sufficient on its own since the effect of the photoperiod depends heavily on the temperature [62,156]. Pitaya grows best at temperatures between 18 and 26 °C, although it can tolerate wider range of 14 to 35 °C [7,157]. However, night-time temperature plays a decisive role: floral differentiation only occurs when minimum temperatures consistently exceed 15 °C [158]. In Mediterranean climates, these limits are usually only reached at the beginning of summer, shifting natural flowering to late June or July (depending on the specific requirements of the cultivars) and consequently placing the harvest period between late July and December. Conversely, prolonged exposure to daytime temperatures above 38 °C can inhibit floral induction and promote bud abscission, thereby impairing fruit set [159,160].

Light intensity also plays an important role. The daily light integral (DLI), which is defined as the total amount of photosynthetically active radiation (PAR, 400–700 nm) received per day (mol m^−2^ day^−1^), has a direct influence on the accumulation of carbohydrate reserves in the cladodes. In pitaya, a minimum DLI of approximately 12 mol PAR m^−2^ day^−1^) is required for commercially viable yields [157]. Adequate solar radiation increases photosynthetic activity, enhancing carbohydrate accumulation in the cladodes. These carbohydrate reserves are essential for supporting the transition from vegetative to reproductive growth, as well as for promoting the initiation of flowering [161]. By contrast, suboptimal light conditions, whether resulting from prolonged cloud cover or excessive shading, reduce the availability of photoassimilates [162], leading plants to maintain vegetative growth and potentially delaying, reducing or even suppressing flowering. Therefore, the transition from vegetative to reproductive growth is promoted by a moderate thermal amplitude, stable warm temperatures and an increase in day length.

In the Mediterranean climate, winter flowering is not possible due to the low temperatures and short days characteristic of this season. The use of supplemental lighting has been explored as a strategy to extend the flowering season and, therefore, sustain high-value fruit production over a longer period [163,164]. Artificial illumination can be implemented through two main approaches: (1) day-length extension, which increases the total duration of light exposure, and (2) night-break (NB) lighting, which temporarily interrupts the continuous dark period [165].

Studies have shown mixed results regarding the efficacy of photoperiod extension on pitaya flowering. Trials conducted in Israel, using high-intensity lamps, showed that day-length extension alone was insufficient to induce flowering [166]. In contrast, research from Taiwan [167] demonstrated that NB treatments of 4–6 h induced excessive off-season flowering, making flower bud thinning necessary. To limit labour costs and ensure high fruit quality, floral bud density was maintained below 50% of the plant’s flowering capacity. A 1 h night interruption achieved this threshold within five weeks in autumn, while in spring, bud thinning enhanced plant responsiveness to a 4 h night-interruption regime applied over four weeks from mid-February, resulting in the earliest fruit harvests. Both experiments were conducted prior to the natural production season and tended to be less effective at higher latitudes with colder winters, since temperature is a critical factor in the regulation of pitaya flowering. Additional evidence [168] indicates that supplementary lighting is only effective when minimum air temperature remains above 15 degrees, reinforcing once again the central role of temperature in the pitaya induction flowering process regulation. Consequently, in Mediterranean regions, the use of photoperiod manipulation to advance or extend the production season is likely to be more effective under protected cultivation systems, where both temperature and light conditions can be controlled.

### 8.3. Temperature Manipulation (Protected Cultivation)

Protected cultivation provides significantly more favourable conditions for floral induction than open-field environments do. The stable thermal regime inside greenhouses or tunnels reduces temperature fluctuations, enabling the minimum night-time temperatures required for flowering to be reached earlier in the season. This allows the induction process to proceed uninterrupted, thus anticipating the onset of flowering and ensuring higher productivity, especially for cultivars with lower light requirements. Consequently, protected systems can extend the flowering and fruiting periods, enabling earlier and later harvests than would be possible outdoors. Extending the production window contributes to greater production consistency and can ultimately increase total yield. However, high summer temperatures (around 38 °C) can inhibit flowering [169]. Since such temperatures are easily exceeded in protected environments, adequate greenhouse ventilation during the summer months is essential to prevent this inhibition.

### 8.4. Water Stress—Regulated Deficit Irrigation

In many tropical and subtropical fruit species, severe or prolonged water stress suppresses floral induction. Conversely, mild or transient stress, such as a temporary reduction in water availability, can redirect assimilates, increase soluble sugar concentrations, and trigger hormonal signals associated with the transition to reproductive growth [170]. Regulated deficit irrigation (RDI), which involves deliberately reducing water supply during specific phenological stages at which mild water stress can be tolerated without compromising yield or fruit quality, is a widely adopted technique in several fruit crops [171,172,173]. RDI can be implemented to save water whilst simultaneously increasing water productivity (more fruit per unit of water used) [174]. This approach also provides greater control over vegetative growth, yield, and fruit size [175].

In the case of pitaya, RDI may offer analogous benefits, particularly given its CAM metabolism, which confers a natural ability to withstand short periods of moderate drought [176,177]. The way pitaya responds physiologically to short-term water deficit provides important insights for irrigation management. According to [177], *S. undatus* maintains a relatively stable stem water status during short-term drought due to its high water-storage capacity. During these periods, cladodes show a gradual decline in water potential yet retain turgor, allowing nocturnal CO_2_ uptake to continue. Once irrigation is restored, both water status and CO_2_ assimilation recover rapidly, demonstrating the plant’s remarkable resilience and minimal physiological impairment. These traits are particularly advantageous in Mediterranean environments, where rainfall is irregular and summer water availability can be limited, reinforcing the potential of pitaya to sustain production under reduced or pulsed irrigation regimes.

Nevertheless, applying RDI to this crop can be more complex. This is attributed to the fact that flowering and fruiting phases frequently overlap during the production season. Reducing irrigation during the pre-flowering period may therefore coincide with stages of fruit enlargement and ripening. Adequate water supply is essential during this stage to maintain fruit size and quality. Therefore, the effective implementation of RDI requires precise timing and should ideally be restricted to the primary floral induction window only, when water restriction may stimulate reproductive signalling without compromising fruit development. Recent molecular findings further support this strategy: a FLOWERING bHLH (FBH) transcription factor, HpbHLH70, was identified in *S. polyrhizus* as being drought-responsive and acting as an accelerator of floral bud induction under water-deficit conditions [153].

Despite its potential, no controlled studies have yet investigated optimal deficit levels or timings for pitaya. This represents an important research gap and an opportunity to develop irrigation strategies aimed at improving water-use efficiency.

### 8.5. Plant Growth Regulators

Plant growth regulators (PGRs) have been investigated as potential tools to manipulate floral induction in pitaya [178], although their effectiveness appears to be strongly cultivar-dependent and is generally less reliable than environmental cues. Giberellins (GA_3_, GA_4_/_7_) typically suppress flowering by promoting vegetative growth, whereas cytokinins (such as 6-benzyladenine and the synthetic cytokinin forchlorfenuron [CPPU; N-(2-chloro-4-pyridinyl)-N′-phenylurea]) enhance floral differentiation when applied prior to the natural induction period. In *S. undatus* and *S. megalanthus*, CPPU advances flowering, and in *S. undatus* it also increases the total number of flowers, while GA_3_ delays flowering and reduces floral yield [166].

Despite this physiological basis, the use of PGRs for flower induction in pitaya remains experimental, and there are no validated, standardised protocols for commercial production. As pitaya cultivation expands into Mediterranean regions, well-designed studies are still needed to determine the optimal timing and dosage of PGRs, as well as their cultivar-specific responses, in order to assess their practical applicability.

## 9. Supplementary Manual Pollination

Pollination is considered one of the most challenging horticultural constraints in pitaya production. In its regions of origin, natural pollination is primarily mediated by nocturnal and diurnal pollinators [144,179]. However, on commercial plantations, the activity of these pollinators is reduced due to habitat fragmentation, resulting in a lower abundance of nocturnal pollinators than in intact ecosystems [180,181]. Additionally, the effectiveness of these agents may be compromised by the heavy rainfall during the flowering period, which can remove pollen from floral structures or induce flower abscission. Furthermore, evidence suggests that both pollen origin and pollen load significantly influence crop productivity and fruit quality [182]. Insufficient pollination frequently results in smaller fruit due to scarce ovule fertilisation, even when fruit set occurs [160]. Several studies in different environmental conditions [38,183] report that most cultivated pitaya genotypes exhibit low fruit development due to self-incompatibility [184], making cross-pollination necessary [185,186]. Morphological characteristics of the flower, such as its size and the clear spatial separation (>2 cm) between male and female organs (herkogamy), reduce the likelihood of contact between reproductive structures, thereby limiting self-pollination [187]. This has been verified in two of the most important cultivars in Iberian commercial plantations: *S. undatus* and *S. costaricencis*. Conversely, this same trait facilitates supplementary manual pollination, which is a common and often necessary practice [188], with the aim of obtaining higher yields and improving fruit quality [189,190].

In the Iberian production regions, natural pollination is presumably limited, with constraints remaining present in terms of self-incompatibility and floral morphology [191]. Previous studies [7] indicate that honeybees (*Apis mellifera*) are the most frequent and efficient diurnal pollinators of pitaya in this region. However, supplementary manual pollination has become a routine and structural practice among farmers, as it eliminates the risks associated with natural pollination and limitations of self-incompatibility [144], despite the increased labour costs involved. This practice involves manually collecting pollen from the stamens and depositing it directly onto the stigma. Several methods of pollen collection can be used. The most traditional approach consists of gently shaking the flower over a container to collect the released pollen, followed by transferring any pollen adhering to the petals into the same container. Another alternative involves the use of small battery-operated vacuum devices. Manual pollination is considered complete when pollen is successfully placed on the stigma, ensuring a higher pollen load than with natural pollination.

In southern Portugal and Spain, pitaya flowers typically reach anthesis at around 11 p.m. during the peak flowering period. [7]. To maximise pollen viability and stigma receptivity, supplementary manual pollination is recommended during or shortly after this period. However, studies report no significant differences between pollination conducted at night and in the early morning. According to Andrade et al. [192], pollen collected at night or in the early morning from *S. undatus* and *S. monacanthus* exhibited high germination capacity. This demonstrates that pollen remains viable throughout anthesis and even during pre-anthesis, enabling successful ovule fertilisation.

Although supplementary manual pollination is effective, several external factors must be considered, particularly the cultivation system and prevailing weather conditions. In open-field production systems, for example, strong nocturnal winds at night can blow pollen out of the flowers, reducing pollination efficiency. Windy conditions also hinder pollen collection and may reduce the amount of pollen available to bees in the early morning. Therefore, when windy nights are anticipated, pollen should be collected as early as possible. Another preventive strategy is to collect surplus pollen and store it in a refrigerator. When stored properly, pitaya pollen can remain viable for up to five days at 4 °C [193] and can be used for subsequent pollinations. For long-term storage of up to nine months, cryopreservation in liquid nitrogen at −196 °C preserves pollen viability and has been associated with an increase in fruit size [144,194].

Manual pollination has become a routine component of pitaya plantation management in the Iberian context, rather than a supplementary or occasional practice. Its integration with cultivar selection, plantation design, and labour organisation is essential to ensuring the economic viability of commercial production.

## 10. Bibliometric Analysis

A bibliometric analysis was conducted to identify research patterns and emerging trends related to cultural practices in pitaya cultivation. The analysis was performed using VOSviewer software (version 1.6.20) to visualise the co-occurrence of keywords extracted from the titles, abstracts and author keywords of scientific publications indexed in the Scopus database. The search covered the period up to 2025, with the earliest records dating back to 1995.

The search strategy applied was as follows: *(“Pitaya” OR “Dragon Fruit”) AND (“Cultural” OR “Agronomic” OR “Cultivation” OR “Practices”) AND NOT (“fungicides” OR “pests” OR “diseases” OR “processing”)*. This search was conducted on 3 January 2026 and yielded a total of 189 relevant documents from multidisciplinary research areas, with a strong emphasis on Agricultural and Biological Sciences.

**Figure 6:**
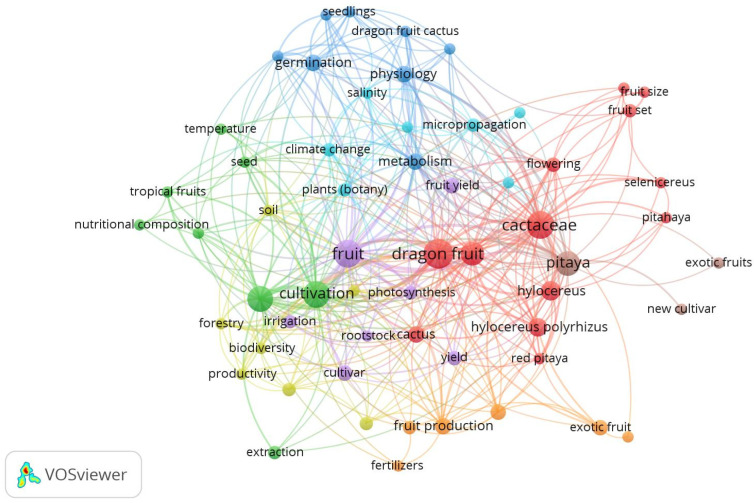
Clustering of the most frequently used words in the titles, abstracts, and keywords of peer-reviewed papers related to cultural practices in pitaya cultivation. The size of the bubbles represents the frequency of occurrence of each term, the thickness of the lines indicates the strength of the co-occurrence links between terms, and the colours denote the different thematic clusters identified by the analysis.

For cluster generation, a minimum threshold of three occurrences per keyword was applied. From a total of 1329 keywords identified, 96 met this criterion and were included in the network analysis. Co-occurrence analysis resulted in 59 items organised into eight distinct clusters, reflecting the main thematic structures of the literature (Figure 6).

The eight clusters can be interpreted as falling within four broader thematic groups. The first group encompasses clusters related to “*pitaya*”, “*dragon fruit*”, the “*Cactaceae*” family, the “*Hylocereus*” genus and cultivar development. This group reflects the taxonomic, botanical and genetic foundations of pitaya research. The clusters in this group are strongly interconnected, with terms such as “*fruit size*”, “*fruit set*”, “*yield*” and “*new cultivar*”, highlighting a research focus on species identification, varietal selection and productivity traits. This indicates that cultivar selection and genetic background are crucial for optimising pitaya adaptation and performance, particularly in non-native growing conditions.

The second group of clusters is dominated by physiological and developmental aspects, such as “*photosynthesis*”, “*metabolism*”, “*flowering*”, “*fruit yield*”, “*salinity*”, “*temperature*” and “*climate change*”. The strong links revealed by these clusters between environmental conditions and plant physiological responses suggest that abiotic factors play a decisive role in pitaya growth and productivity. The prominence of these terms indicates a growing interest in understanding how pitaya respond to stress conditions, which is especially relevant in Mediterranean environments, characterised by high irradiance, seasonal drought and temperature fluctuations.

The third group integrates clusters related to propagation, early growth and plant establishment, such as “*seedlings*”, “*germination*”, “*micropropagation*”, “*seed*” and “*rootstock cactus*”. These clusters reflect research efforts aimed at improving propagation techniques and nursery practices to ensure uniformity and vigour and to facilitate successful field establishment. The presence of micropropagation and rootstock-related terms highlights the importance of alternative propagation strategies to overcome limitations related to the availability of plant material and its ability to adapt to diverse soil and climatic conditions.

The fourth group comprises clusters that focus on agronomic management and production systems, including “*cultivation*”, “*irrigation*”, “*soil management*”, “*fertiliser use*”, “*productivity*”, “*biodiversity*” and “*forestry*”. These clusters emphasise cultural practices and resource management strategies that directly influence yield and sustainability. The connections with broader agroecological concepts suggest a growing interest in integrating pitaya into diversified and sustainable cropping systems rather than treating it solely as a niche exotic fruit crop.

Overall, the bibliometric analysis indicates that, to further expand pitaya cultivation in the Iberian Peninsula, greater research efforts are needed on irrigation management under water-limited conditions, nutrient management tailored to Mediterranean soils, pruning and canopy management to optimise light use, and the selection of cultivars and rootstocks adapted to abiotic stresses, particularly drought, salinity and high solar radiation.

## 11. Final Remarks

At an agronomic level, pitaya represents a highly attractive crop for farmers, as it reaches commercial production relatively quickly, achieves satisfactory productivity, and allows small, cultivated areas to be economically viable. These characteristics result in a relatively rapid economic return and contribute to farm diversification, particularly for producers engaged in organic farming or considering conversion to organic production. In addition, pitaya offers significant potential for value enhancement in niche markets, such as organic and functional foods, as it remains associated with an expanding and dynamic market. Nevertheless, farmers considering pitaya cultivation should undertake it only when supported by robust and well-substantiated prospects for effective fruit marketability.

Under the climatic conditions of the Iberian Peninsula, the appropriate combination of agronomic practices should enable successful pitaya cultivation by achieving good yields, particularly in protected cultivation systems. From a practical perspective, the adoption of integrated and sustainable management approaches, including efficient irrigation strategies, soil conservation practices, and appropriate vigour control, is essential to ensure hight productivity, fruit quality, and environmental sustainability. The versatility of pitaya further supports its integration into diversified agroecosystems and low-input production systems.

Future research should prioritise multi-year field trials comparing different production systems, with integrated assessments of plant performance, yield, and fruit quality under Mediterranean conditions. Such efforts will be crucial for developing robust, regionally adapted guidelines and for supporting the long-term sustainability of pitaya cultivation in the Iberian Peninsula.

## Figures and Tables

**Table 1:** Summary of pitaya maintenance pruning operations according to timing in the production cycle.

Pruning Time	Pruning Operations
Before flowering (two months prior)	Removal of unproductive, weak, or poorly positioned cladodes. Elimination of vegetative shoots from lateral buds. Selection of few well-positioned shoots to form future production cladodes. Removal of cladodes affected by pests and/or diseases.
After harvest (after two weeks)	Removal of old, shaded cladodes as well as those that have already exhibited high productivity. Reduction of canopy density to improve ventilation and illumination. Selection of well-positioned shoots for the next cycle’s productive cladodes.
Throughout the year	Removal of shoots and young cladodes that are unsuitable for developing into productive cladodes. Removal of cladodes affected by pests and/or diseases.

## Data Availability

No new data were created or analyzed in this study. Data sharing is not applicable to this article.
